# Impact of a resident and student-led video visitation navigation program

**DOI:** 10.1186/s12909-022-03172-6

**Published:** 2022-02-19

**Authors:** Rachel Yang, Smitha Ganeshan, Avery Thompson, Sophie McAllister, Michelle Mourad

**Affiliations:** 1 School of Medicine, University of California San Francisco, San Francisco, CA USA; 2 Department of Medicine, University of California San Francisco, San Francisco, CA USA

**Keywords:** Telehealth, Patient support persons, Trainee engagement, Quality improvement, COVID-19

## Abstract

**Background:**

Many institutions implemented telehealth initiatives to provide social support for patients during the SARS-CoV-2 (COVID-19) pandemic. Little is known about the impact of these programs on patient support persons and the trainees who facilitated them.

**Objective:**

To assess perceptions of a resident physician and medical student-driven video visit program.

**Methods:**

We designed and implemented a trainee-led video visit navigation program across three affiliated urban hospitals to facilitate video visits between patients and their support persons. We used descriptive statistics to understand the patient population served by the program and employed surveys for support persons and trainees to assess attitudes on the program.

**Results:**

From April to June 2020, a total of 443 video visits were completed. Surveys were conducted for 101 out of 184 (54.9%) support persons and 39 out of 65 (60.0%) of medical trainees. Surveys demonstrated that video visits helped alleviate the stress and anxiety of support persons having a hospitalized loved one they could not visit. For trainees, facilitating these connections helped mitigate stress and provided a mechanism to contribute to the pandemic response.

**Conclusion:**

Telehealth navigation programs provide high levels of connection for patients and their support persons during the COVID-19 pandemic and potentially beyond. Residents and medical students involved in these initiatives mobilized telehealth modalities to improve experiences with care delivery.

**Supplementary Information:**

The online version contains supplementary material available at 10.1186/s12909-022-03172-6.

## Background

Visitor restrictions designed to limit the spread of SARS-CoV-2 (COVID-19) isolated patients from their support persons [1]. The inability to directly communicate with loved ones caused distress for both patients and their support persons and limited caregiver participation in shared decision-making. These experiences also engendered moral distress among frontline healthcare providers, including resident physicians and students, who witnessed the isolation and endeavored to fill the gaps [2].

In response to visitor restrictions, many hospitals implemented telehealth initiatives designed to facilitate patient connection to outside support persons through video visits [3–7]. However, limited data exists on the impact of telehealth initiatives on support persons and the providers who facilitated visits. One virtual program adopted in an intensive care unit found high levels of support and approval among family members, who reported feelings of gratitude, relief, and a sense of closure for those who lost loved ones [8]. Another program focusing on virtual visits for patients with COVID-19 found that while responses from relatives were overall positive, staff facilitating these calls experienced emotional strain from seeing distressed support persons over video [9].

At our academic medical centers, a group of medical students and resident physicians implemented an inpatient telehealth program to connect patients and support persons through video visits. In this study, we report on the characteristics of patients served by the video program intervention, as well as the impact on patients’ support persons and the trainees who implemented the program.

## Methods

### Study site and program overview

The video visit navigation program was designed in March 2020 by a group of resident physicians, medical students, and faculty at affiliated teaching hospitals where medical trainees participate in clinical rotations. The institutions include a tertiary and quaternary referral center, a community safety net hospital, and a federal healthcare facility.

Trainees used a rapid quality improvement approach to design the video visit navigation program aimed at facilitating video visits between patients and their support persons [3, 10]. Trainees created a consultative service staffed by medical student volunteers, who fielded referrals from clinicians of patients who would benefit from a video visit with their support persons. Trainees facilitated video visits between patients and support persons by scheduling an appropriate time, assisting support persons with downloading and troubleshooting secure video-conferencing software (Zoom Meetings, Zoom Video Communications), and providing tablets and ancillary hardware to patients’ rooms. Patients and their support persons could use the service for future visits as needed. The program also provided clinicians with access to tablets for on-demand video visits after hours.

### Study population

We identified all patients and their support persons who participated in a video visit from April 13, 2020, to May 31, 2020, at all three clinical sites. We collected demographic and clinical data of all patients who participated in video visits via chart review. Patients with missing medical record number data or restricted medical records were excluded from the analysis. COVID-19 positive patients were not represented in the analysis, as hospital administration restrictions at the time of this initiative precluded medical students from engaging in the care of and facilitating video calls for COVID-19 positive patients. Contact information of patient support persons was collected as part of the video visit intervention. Support persons without recorded contact information were excluded from the study. The trainees involved in facilitating video visits were identified through a communication platform used during the navigation program operations.

### Survey instruments

We developed two survey instruments, one for support persons who participated in the visits and one for the trainees who facilitated them. The development of both surveys was informed by focus groups and interviews with physicians and by a systematic review of prior surveys to assess patient and clinician perspectives on telemedicine satisfaction [11–13]. As no published surveys to our knowledge directly assess perspectives on video visits that connect patients with their support persons, the wording of survey questions was adjusted to this specific context of video visits.

The support person survey assessed the impact of video visits on support persons’ feelings of isolation and stress. Support persons rated their agreement with statements regarding their perspectives on visitor restrictions and the video visit navigation program on a Likert scale from 1 (strongly disagree) to 5 (strongly agree) (Appendix A). The survey for patient support person was developed by the investigators with guidance from a panel of physicians with expertise in patient experience. The surveys were piloted among a group of attending physicians and adjustments were made based on feedback regarding the flow, length, clarity, and content validity.

Trainees also provided their perspectives on visitor restrictions and the impact of the video visit navigation program on connection and emotional distress using a Likert scale from 1 (strongly disagree) to 5 (strongly agree) (Appendix B). Trainees additionally commented on their general experience with the navigation program through open-ended survey questions. The survey of medical trainees was created to have comparable components as the patient support person survey. The development of the survey was also informed by pilot testing among trainees who reviewed the survey language for flow, length, clarity, and content validity.

### Survey process

We contacted patient support persons, the primary recipient of video visits, by phone a maximum of two times to administer the phone-based survey. Support persons provided verbal informed consent during the survey for this minimal risk study. Trainees who facilitated the video visits were contacted by email with an online survey. A total of two reminder emails were sent to each trainee.

### Data analysis

Patient demographics were summarized using descriptive statistics. Continuous variables were reported as median and interquartile range. Categorical variables were reported as number and percent of total. Survey questions that were not answered or skipped by respondents were omitted from the analysis. Fisher exact test at a significance level of 0.05 was used to compare categorical variables. Qualitative responses in the trainee survey were analyzed using a rapid qualitative template analysis which included themes from the Theory of Planned behavior (e.g., attitudes, beliefs, subjective norms, perceived behavioral control, behavioral intention). Templates were iteratively revised to incorporate emergent themes [14]. Two reviewers independently coded each survey response and discussed discrepancies to achieve greater than 95% concordance. Key themes and quotes were extracted and collated for analysis. Representative quotes were edited to exclude or modify identifiable patient or trainee details.

## Results

**Fig. 1:**
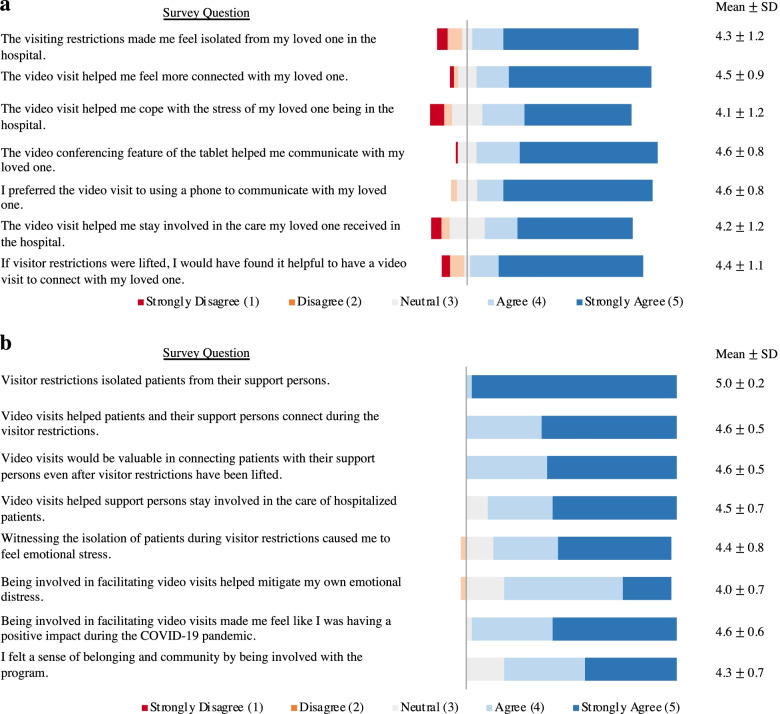
**a**, Survey responses of support person perceptions of video visits; **b**, Survey responses of medical trainee perceptions of facilitating video visits

During the seven-week period from April 13, 2020, to May 31, 2020, trainees facilitated a total of 443 video visits for 184 unique patients from a range of referring services. The characteristics of the video visit participants are summarized in Table 1. Over half of the patients, 97 (52.7%) had repeat visits. The clinical acuity of patients was high: 97 patients (52.7%) required intensive care unit (ICU) level of care, 42 (22.8%) received a palliative care consult, and 25 (13.6%) died during the hospitalization. Fig. 1 summarizes the responses of the patient support persons and medical trainees to the surveys.

**Table 1:** Demographics of video visit participants

Demographic characteristics	
Number of video visits	443
Total number of patients with video visits	184
Number of patients with repeat visits	97 (52.7%)
Median age of patients	70 (IQR 24.5)
Admitting service	
Medicine	107 (58.2%)
Surgery	50 (27.2%)
Neurology	11 (6.0%)
Cardiology	10 (5.4%)
Psychiatry	3 (1.6%)
Skilled Nursing Facility	3 (1.6%)
Patient preferred language	
English	130 (70.7%)
Spanish	19 (10.3%)
Cantonese	18 (9.8%)
Tagalog	5 (2.7%)
Toisanese	4 (2.2%)
Other	8 (4.3%)
Number of patients who received Palliative Care consult	42 (22.8%)
Number of patients who received ICU level care	97 (52.7%)
Median length of hospital stay	13 (IQR 18)
Number of patients who died while hospitalized	25 (13.6%)
Number of support persons surveyed	101
Median number of support persons per visit	3 (IQR 3)
Support person's relationship to patient	
Immediate family member	88 (87.1%)
Extended amily Member	11 (10.9%)
Friend	2 (2.0%)
Median distance support person lived from hospital (miles)	5.1 (IQR 38)
Support person preferred language	
English	91 (90.1%)
Spanish	6 (5.9%)
Cantonese	1 (1.0%)
Other	3 (3.0%)

### Patient support person survey

Of the 184 support persons that participated in video visits, 101 (54.9%) completed the survey, 58 individuals could not be contacted or did not answer the phone call, and 25 refused the survey. Of the patient support persons who completed the survey, 88 (87.1%) were immediate family members.

Patient support persons agreed or strongly agreed (*n* = 80, 82.5%) that the visiting restrictions isolated them from patients in the hospital, but they felt that the video visit helped them connect (*n* = 86, 86.9%) and cope with the stress of their loved one being in the hospital (*n* = 74, 74.0%). A smaller proportion agreed or strongly agreed that the video visit helped them stay involved in the care of the hospitalized patient (*n* = 72, 73.5%). Support persons reported a preference for video visits over using a phone (*n* = 87, 87.0%) and endorsed the continuation of video visit when visitor restrictions are lifted (*n* = 85, 85.9%). There were no significant differences in the proportion of support persons of patients who received ICU level of care compared to the support persons of patients who did not receive ICU level of care who agreed or strongly agreed with all statements in the survey.

### Medical trainee survey

Of the 65 medical trainees involved in the initiative, 39 (60.0%) completed the survey. One trainee who could not be contacted by email and five trainees who submitted surveys with empty responses were not included in the analysis. Survey respondents included 31 (79.5%) medical students and 8 (20.5%) resident physicians.

Trainees (*n* = 39, 100%) agreed or strongly agreed that the visitor restrictions isolated patients from their support persons, but they felt that video visits helped connect patients to their support persons (*n* = 39, 100%) and helped support persons stay involved with care of hospitalized patients (*n* = 35, 89.7%). Trainees involved in the program reported high levels of emotional distress due to visitor restrictions during the pandemic: 33 (84.6%) trainees agreed or strongly agreed that witnessing the isolation of patients during visitor restrictions was stressful. Trainees felt they were having a positive impact during the pandemic through their involvement in the initiative (*n* = 38, 97.4%) and felt that being a part of the program engendered a sense of belonging and community (*n* = 32, 82.1%). There were no significant differences in the proportion of medical students compared to residents who agreed or strongly agreed with all statements in the survey.

### Narrative description of video visit navigation

**Fig. 2:**
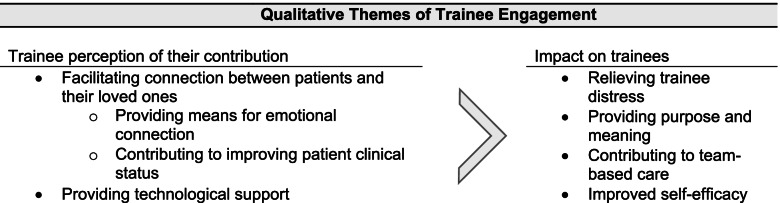
Schema of key themes from qualitative interviews of medical trainees

Surveyed trainees highlighted the impact of the video visit navigation program on patients and support persons as well as themselves (Table 2, Fig. 2). Respondents reported that the video visit navigation program fostered positive feelings between patients and their support persons:

*“I helped a patient in the skilled nursing facility who hadn’t seen his spouse for at least 4 weeks. When I walked in the room, he was lying in bed, still, and looked like he was in pain. When we got video of his spouse, he immediately burst into tears and they spoke for an hour. When I came back to the room, he was sitting up in bed, smiling, laughing.”*

**Table 2:** Qualitative themes and representative quotes from survey of medical trainees

Theme	Representative Quote(s)
Facilitating positive emotional connection for patients and their support persons	•*"One elderly man had a video visit with his wife on several days over the course of 2 weeks. The amount of love with which he spoke to her was very touching."* •*"A stroke recovery patient hadn't been able to form words, but after connecting her with her daughter through video, she formed words. It was very emotional and meaningful."*
Allowing meaningful support for patients with critical illness	•*"I helped facilitate a video visit for one of the ICU patients who had poor prognosis. Even throughout the video visit, the patient was not able to participate due to altered mental status. It was heartbreaking to see how his family could not be physically present as he was going through very challenging time. Still, his family was very grateful to be able to see him regularly given the circumstances. It definitely showed how valuable this program is in connecting patients with their loved ones."* •*"I was also moved by how video calls at end of life facilitated spiritual tradition and community. Allowing video calls at end of life allowed people to bring in family and spiritual leaders from around the world. We connected with callers from many different countries. We communicated with nuns and priests who were themselves locked down in a congregate setting and were able to provide last rights over the video call."*
Promoting improved patient clinical status and care	•*"I also saw so many patients perk up and their physicians were surprised by how sharp they were; we noticed people’s mental status improved with human connection."*
Supporting patients with limited digital literacy and/or access	•*"I remember a man who suffered a disabling stroke and video chatted with his family several times a week. Because of his physical deficits he was unable to use a phone independently and greatly benefited from having a volunteer at bedside to set up the tablet and get the call started. I worry about how he will be able to connect with his family without people proactively approaching the patient and physically helping with the call."* •*"There was a large range of tech skill in the people I talked to and there is definitely need for tech support in order to train some families on how to use video conferencing software."*
Relieving provider distress	•*"I found it very challenging to bear witness to such suffering and isolation. We always find it humbling to be in the room during these key moments such as birth and death. To be the one in the room when the patient was dying and their own children couldn't be there, that was another level."*
Providing meaning and purpose to the work of medical trainees	•*"It had a tremendous impact on me- it felt like we were honoring patients' humanity when so many things were limited by COVID. We were all paying a steep price as a society for the sake of public health, but, as with many things, the biggest burden fell on the most vulnerable who need their families to advocate for them."* •*"It felt lovely to feel like I was helping—patients, who must have felt so alone, and the care team, especially nurses, who had to witness so much suffering each day"*
Demonstrating teamwork in medicine	•*"It shows what sheer determination and incredible teamwork can do."*

In addition, the video navigation program allowed support persons to engage with patients who were critically ill or at the end of life:

*“One patient who was comatose and being moved to comfort care had family members across the country singing songs, praying, and sharing memories- holding a digital vigil for hours during her final days.”*

Other experiences suggested an impact of video visit navigation on patients and support persons’ physical wellbeing.

*“For one patient, the nurses told us that the patient's condition started improving when she was able to talk to her husband every day because of the [video] visits we facilitated.”*

Trainees facilitating video visits commented that the video visit program benefitted patients and support persons with limited ability to use digital technologies.

*“It helped me realize how video conferencing applications can be confusing to people not used to it, and it challenged my assumptions that most people have adequate technology/video access/ability to understand how to use technology.”*

Witnessing the connection enabled through video visit navigation had impact on the trainees who were involved in facilitating visits by mitigating emotional distress:

*“It was both emotionally distressing seeing the isolation patients were feeling and incredibly rewarding to help mitigate that.”*

The project helped give trainees a sense of meaning and purpose in their work.

*“It was beneficial to know that as a medical student I could still make a difference in patient care even while off the wards. It was a reminder of how medicine is more than just looking at labs and making a diagnosis, but there’s a very human aspect that is profound and few things can replace it.”*

The project also highlighted the importance of teamwork in medicine.

*“It made me think about all the moving pieces in healthcare and how even if each person may have a different role, each contribution is valuable since no one person can do everything.”*

## Discussion

The COVID-19 pandemic separated hospitalized patients from their support persons causing emotional distress and social isolation. Our results suggest that an inpatient video visitation consult service using video visits to connect hospitalized patients with their support persons is both feasible and valuable. Video visits alleviated the stress and anxiety of support persons having a hospitalized loved one they could not visit. Notably, we found that for trainees, facilitating these connections helped alleviate their stress and provided a means of contribution to the pandemic response.

Over half of the video visits involved repeat visits, which in conjunction with the positive responses to the survey suggests satisfaction with the service. Support persons indicated that they would find a video visit service useful even in the absence of hospital visitor restrictions, which highlights the opportunity to improve support person engagement through telehealth infrastructure. Previous studies also show that separating families from the patients can adversely impact patients’ feelings of security and their health outcomes [15]. In a previous study of critically ill family members, families believed they played a critical role as an advocate, defender and coach, ensuring safe, appropriate and effective care [16]. Our finding that separation of support persons from their hospitalized family and friends was a source of isolation and stress is thus consistent with these findings and those at other institutions [2, 17].

To support patients and remain compliant with social distancing and infection control best practices, institutions replaced in-person visits with virtual ones. The published literature has focused on the technology and workflow related to these visits, while fewer studies have examined the impact of virtual visitation on families or providers, including trainees. Similar to our study, two other studies have found that video visits were associated with positive emotions for loved ones like happiness, hope and gratitude [9, 18].

In addition to the impact of the inpatient telehealth program on support persons, the video visit program had a strong positive impact on the trainees involved with this initiative who reported that their involvement helped mitigate stress. These findings are not universal among health care workers: one study found that 23% of surveyed staff involved in video visits had negative responses relating to emotional strain, difficult situations, the sadness of the visits, and communication with ward staff [9]. Our differing results may stem from the fact that trainees, both resident physicians and students, were initially excluded from the care of COVID-19 positive patients. Particularly for medical students whose participation in clinical care was limited following the American Association of Medical College (AAMC) guidance in March 2020 [19], this program provided the opportunity to develop new care systems that directly addressed the changing healthcare environment during COVID-19 [20, 21]. Literature suggests that participation in improvement interventions can be a meaningful activity for trainees and provides an opportunity to improve care and develop leadership skills [22–24]. Through such initiatives, resident physicians can create innovative programs and learning opportunities that can mobilize and engage undergraduate medical learners. Creating a supportive environment for entrepreneurial initiatives can improve operations on the frontlines, create new learning opportunities, and engender feelings of self-efficacy.

In this analysis, the direct perspectives and experiences of the patients who were isolated from their support persons were not assessed. Since many patients who had a video visit passed away or suffered from medical co-morbidities (e.g., intubated, experiencing delirium) that limited their ability to participate in surveys, we chose to investigate the perspectives of the other important people directly involved in the video visits: the patient support persons who were recipients of the video visits and the medical trainees who facilitated the video visits. Both patient support persons and medical trainees had high levels of agreement that the video calls helped provide meaningful connection during visitor restrictions. While the isolation of patients in the context of visitor restrictions was distressing for all, participating in this video visit initiative helped alleviate this stress for both the support persons who connected with their loved ones and the trainees who facilitated this connection.

While 89.7% of medical trainees agreed or strongly agreed that the video visits helped loved ones stay involved in the care of hospitalized patients, only 73.5% of loved ones of patient support persons agreed or strongly agreed that the video visit helped them stay involved in the care their loved one received in the hospital. Given that this initiative was designed primarily with the goal of bridging connection between patient and their support persons rather than between support persons and patient care teams, further design considerations could be implemented to enhance and better incorporate the communication of care planning to remote patient support persons.

Our program demonstrates the ability of trainees to creatively organize a volunteer workforce and mobilize the support of the community around enhancing patient care. Our entirely volunteer workforce received no compensation, and the operational costs were entirely funded by donations. Our team solicited tablet donations, utilized free video conferencing software, and fundraised around $10,000 to pay for ancillary hardware (e.g., speakers, tablet stands, and cases). In June of 2020, at our main hospital the Telemedicine Resource Center (TRC) assumed responsibility for staffing the video visit service supporting two full-time equivalent clerk positions. The TRC also integrated consult ordering workflows into the electronic medical record, allowing providers to order a video visit within the electronic medical record seven days a week from 8 AM – 8 PM. The success of the program and the responses of our trainees suggest that medical trainees are suited to mobilize resources and advocate for sustainable improvements in gaps of care delivery, which may serve as a powerful antidote for their own anxiety and stress.

Limitations of this study include response bias since support persons with strongly negative or positive experiences may have been more likely to engage in the survey. Consults to the video visit service were initiated by providers, which may pose a source of selection bias of the video visit participants. Additionally, survey administrators also led tablet operations, which may introduce acquiescence bias for the support persons who engaged in video visits as well as the trainees who facilitated the program. Lastly, patient experience and satisfaction were not directly assessed in this analysis.

## Conclusion

This study demonstrates the meaningful benefits a telehealth initiative had on engaging trainees and connecting patients with their outside support systems across diverse practice settings. Engaging trainees directly in times of crisis may foster a sense of meaning, purpose, and community that can lead to sustainable improvements in the healthcare delivery system.

## Supplementary Information

**Additional file 1. **

## Data Availability

The datasets used and/or analyzed during the current study are available from the corresponding author on reasonable request.

## Abbreviations

AAMC: American Association of Medical College
COVID-19: SARS-CoV-2
ICU: Intensive Care Unit
TRC: Telemedicine Resource Center
