# Supplementary material for: Impact of a resident and student-led video visitation navigation program
Source: BMC Med Educ. 2022 Feb 19;22:110. doi: 10.1186/s12909-022-03172-6 (PMC8857532; doi:10.1186/s12909-022-03172-6)
Supplement: Supplementary file 1 — Additional file 1. [file 12909_2022_3172_MOESM1_ESM.docx]

Appendix A: Support Persons Survey

1. On a scale of 1-5 with 1 being strongly disagree, 2 being disagree, 3 being neither disagree or agree, 4 being agree, and 5 being strongly agree, what is your level of agreement with each statement.
   1. The visiting restrictions made me feel isolated from my loved one in the hospital.
   2. The video visit helped me feel more connected with my loved one.
   3. The video visit helped me cope with the stress of my loved one being in the hospital.
   4. The video conferencing feature of the tablet helped me communicate with my loved one
   5. I preferred the video visit to using a phone to communicate with my loved one.
   6. The video visit helped me stay involved in the care my loved one received in the hospital.
   7. If visitor restrictions were lifted, I would have found it helpful to have a video visit to connect with my loved one.
2. Do you have any additional comments or feedback?

Appendix B: Medical Trainee Survey

1. On a scale of 1-5 with 1 being strongly disagree, 2 being disagree, 3 being neither disagree or agree, 4 being agree, and 5 being strongly agree, what is your level of agreement with each statement.
   1. Visitor restrictions isolated patients from their support persons
   2. Video visits helped patients and their support persons connect during the visitor restrictions
   3. Video visits would be valuable in connecting patients with their support persons even after visitor restrictions have been lifted.
   4. Video visits helped support persons stay involved in the care of hospitalized patients.
   5. Witnessing the isolation of patients during visitor restrictions caused me to feel emotional stress.
   6. Being involved in facilitating video visits helped mitigate my own emotional distress.
   7. Being involved in facilitating video visits made me feel like I was having a positive impact during the COVID-19 pandemic.
   8. I felt a sense of belonging and community by being involved with the program.
2. Please share any stories of facilitating video visits that resonate with you, including patient stories or comments about being involved with this initiative.
3. What impact did facilitating video visits have on you?
